# Tumor Growth Rate Estimates Are Independently Predictive of Therapy Response and Survival in Recurrent High-Grade Serous Ovarian Cancer Patients

**DOI:** 10.3390/cancers13051076

**Published:** 2021-03-03

**Authors:** Thomas Bartl, Jasmine Karacs, Caroline Kreuzinger, Stephanie Pfaffinger, Jonatan Kendler, Cristina Ciocsirescu, Andrea Wolf, Alexander Reinthaller, Elias Meyer, Maximilian Brandstetter, Magdalena Postl, Eva Langthaler, Elena Braicu, Ignace Vergote, Paula Cunnea, Charlie Gourley, Wolfgang D. Schmitt, Dan Cacsire Castillo-Tong, Grimm Christoph

**Affiliations:** 1 Department of Obstetrics and Gynecology, Division of General Gynecology and Gynecologic Oncology, Comprehensive Cancer Center, Medical University of Vienna, 1090 Vienna, Austria; thomas.bartl@meduniwien.ac.at (T.B.); crciocsiresc@edu.aau.at (C.C.); alexander.reinthaller@meduniwien.ac.at (A.R.); m.brandstetter@salk.at (M.B.); magdalena.postl@meduniwien.ac.at (M.P.); christoph.grimm@meduniwien.ac.at (G.C.); 2 Translational Gynecology Group, Department of Obstetrics and Gynecology, Comprehensive Cancer Center, Medical University of Vienna, 1090 Vienna, Austria; jasmine.karacs@meduniwien.ac.at (J.K.); caroline.kreuzinger@ist.ac.at (C.K.); stephanie.koller@chello.at (S.P.); kendler.jonatan@gmx.at (J.K.); andrea.wolf@meduniwien.ac.at (A.W.); 3 Center for Medical Statistics, Informatics and Intelligent Systems, Medical University of Vienna, 1090 Vienna, Austria; elias.meyer@meduniwien.ac.at; 4 Department of Pathology, Medical University of Vienna, 1090 Vienna, Austria; eva.langthaler@neduniwien.ac.at; 5 Tumor Bank Ovarian Cancer Network, Department of Gynecology, Charité Universitätsmedizin Berlin, 10117 Berlin, Germany; Elena.Braicu@charite.de; 6 Department of Gynecologic Oncology, Leuven Cancer Institute, University Hospitals Leuven, KU Leuven, 3000 Leuven, Belgium; ignace.vergote@uzleuven.be; 7 Ovarian Cancer Action Research Centre, Department of Surgery and Cancer, Imperial College London, London W12 0HS, UK; p.cunnea@imperial.ac.uk; 8 Nicola Murray Centre for Ovarian Cancer Research, Cancer Research UK Edinburgh Centre, MRC IGMM, University of Edinburgh, Western General Hospital, Edinburgh EH4 2XR, UK; charlie.gourley@ed.ac.uk; 9 Charité-Universitätsmedizin Berlin, Corporate Member of Freie Universität Berlin, Humboldt-Universität zu Berlin and Berlin Institute of Health, Institute of Pathology,10117 Berlin, Germany; wolfgang.schmitt@charite.de

**Keywords:** ovarian cancer, therapy response, platinum-resistant, recurrence, growth rate

## Abstract

**Simple Summary:**

While latest evidence suggests that some patients with recurrent high-grade serous ovarian cancer may profit from reinduction with platinum-based chemotherapy regimens, the selection of patients who are likely to respond remains difficult. The present study therefore aimed to adapt a mathematical model, which used frequently available laboratory values to estimate growth rates of recurring tumors as an objectifiable surrogate of both therapy response and patient survival. After clinical validation, the model may help to personalize treatment strategies and thereby increase survival of affected patients.

**Abstract:**

This study aimed to assess the predictive value of tumor growth rate estimates based on serial cancer antigen-125 (CA-125) levels on therapy response and survival of patients with recurrent high-grade serous ovarian cancer (HGSOC). In total, 301 consecutive patients with advanced HGSOC (exploratory cohort: *n* = 155, treated at the Medical University of Vienna; external validation cohort: *n* = 146, from the Ovarian Cancer Therapy–Innovative Models Prolong Survival (OCTIPS) consortium) were enrolled. Tumor growth estimates were obtained using a validated two-phase equation model involving serial CA-125 levels, and their predictive value with respect to treatment response to the next chemotherapy and the prognostic value with respect to disease-specific survival and overall survival were assessed. Tumor growth estimates were an independent predictor for response to second-line chemotherapy and an independent prognostic factor for second-line chemotherapy use in both univariate and multivariable analyses, outperforming both the predictive (second line: *p* = 0.003, HR 5.19 [1.73–15.58] vs. *p* = 0.453, HR 1.95 [0.34–11.17]) and prognostic values (second line: *p* = 0.042, HR 1.53 [1.02–2.31] vs. *p* = 0.331, HR 1.39 [0.71–2.27]) of a therapy-free interval (TFI) < 6 months. Tumor growth estimates were a predictive factor for response to third- and fourth-line chemotherapy and a prognostic factor for third- and fourth-line chemotherapy use in the univariate analysis. The CA-125-derived tumor growth rate estimate may be a quantifiable and easily assessable surrogate to TFI in treatment decision making for patients with recurrent HGSOC.

## 1. Introduction

Despite the recent introduction of new targeted therapies for high-grade serous epithelial ovarian cancer (HGSOC), platinum-based chemotherapy regimens remain the cornerstone of primary and recurrent HGSOC. Platinum-based therapies achieve primary response rates around 80%, and they have an attenuated efficacy during relapse. Patients enter a cycle of recurrences and remissions, with the response rates and time intervals between recurrences generally decreasing, until the patient dies of their disease [1]. Therefore, research has focused on optimizing the sequences and combinations of available drugs, which has significantly improved HGSOC outcome in recent years. The use of platinum-based regimens in patients with recurrent non-platinum-naïve tumors has been standard practice for the past two decades. Clinical observations of responses after reinduction have challenged the classical oncologic concept of acquired cellular platinum resistance due to Darwinian selection [2].

To resolve this issue, a clinical surrogate of therapy response was proposed, which would predict whether patients would respond to platinum again. The so-called platinum-free interval (PFI), defined as the time between the last administration of platinum-based chemotherapy and diagnosis of cancer recurrence, was implemented in three relatively small studies without special attention to tumor markers in an era when recurrence was mostly diagnosed radiologically and/or clinically [3,4,5]. These previous studies have significantly shaped the perception of “clinical” platinum resistance and definition of subsets of patients with HGSOC; these have been adopted in recent years. In a previous study, patients with a therapy-free interval (TFI) <1 month, <6 months, and between 6 and 12 months were classified as platinum-“refractory,” platinum-“resistant,” and “partially sensitive” to platinum, respectively. Many clinical trials have adopted this classification. At the fifth Ovarian Cancer Consensus Conference in Tokyo (2015), PFI was considered as a TFI of platinum-based chemotherapy (TFIp) that helped to classify patients as “potentially benefiting from platinum-based chemotherapy” or “potentially not benefiting from platinum-based chemotherapy.” To date, there is no widely recognized predictive model to facilitate the estimation of platinum response. Although cancer antigen (CA-125) has been shown to have predictive value, all available studies are short-term models with absolute cut-offs or doubling times [6]. A re-evaluation of CA-125 with newer quantifiable statistical methods could help create more personalized strategies in predicting platinum response and to allow for correct allocation of platinum to only those patients who will derive actual treatment benefit, even second or third line. Therefore, this was conducted to assess the predictive and prognostic value of CA-125, based on tumor growth rate estimates in patients with recurrent HGSOC.

## 2. Materials and Methods

This study was a retrospective exploratory data analysis of patients with HGSOC, who underwent primary cytoreductive surgery for advanced-stage disease (International Federation of Gynecology and Obstetrics (FIGO) IIB–IV). All consecutive patients who received both primary and recurrence therapy at the Department of Obstetrics and Gynecology of the Medical University of Vienna between 2000 and 2015 and who were followed up for at least 60 months comprised the exploratory cohort. The study was conducted in accordance with the Declaration of Helsinki ethical guidelines and approved by the local ethics committee (EK1966/2020). Informed consent was obtained from all the patients prior to sample collection.

Previously published data of the Ovarian Cancer Therapy–Innovative Models Prolong Survival (OCTIPS) consortium of patients undergoing primary debulking surgery followed by platinum-based chemotherapy between 2000 and 2013 at the Department of Gynecology, Charité Berlin, Germany; Department of Gynecologic Oncology, Leuven, Belgium; Department of Surgery and Cancer, Imperial College London, London; and the Centre for Ovarian Cancer Research University of Edinburgh, Western General Hospital, Edinburgh, UK comprised the validation cohort. In total, 155 patients comprised the exploratory cohort, and 146 patients comprised the validation cohort. The exclusion criteria were: Patients <18 years at the time of primary therapy, patients with concomitant malignancies, patients who received neoadjuvant therapy, or patients who received oncologic therapy apart from cytotoxic chemotherapy and/or bevacizumab, such as poly (ADP-ribose) polymerase inhibitors.

All cytoreductive surgeries were conducted by trained gynecologic oncology surgeons. After surgery, adjuvant platinum-based combination chemotherapy with or without bevacizumab was administered to all patients. Recurrence therapy consisted of cytotoxic chemotherapy with or without platinum or bevacizumab, as indicated in the current evidence.

Overall survival (OS) was calculated as the time between primary cytoreductive surgery and the date of death or last follow-up. The TFI was calculated as the time between the last therapy dose and recurrence, as documented in the patients’ charts. The therapy response was classified as a partial response or complete response, as documented in the patients’ charts. 

**Figure 1:**
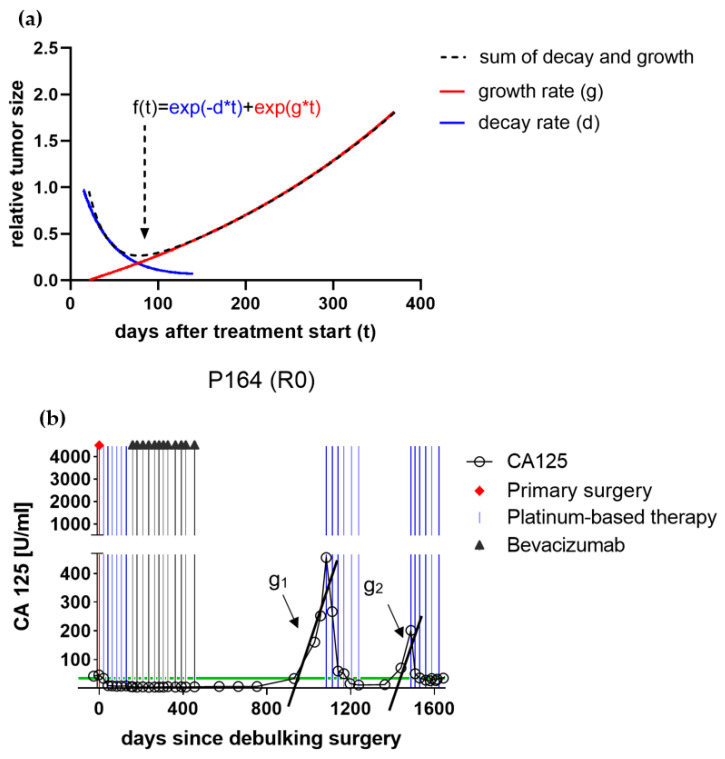
(**a**) The plotted two-phased mathematical equation f(t)=exp(−d×t)+exp(g×t)−1 was proposed by Stein et al. to reflect the sum of decay and subsequent growth of a recurrent solid tumor under therapy. An initial logarithmic decay may shrink the relative tumor size to subclinical dimensions. As soon as cell growth outnumbers decay, the tumor may re-grow to a clinical recurrence. The assumed tumor growth rate (g) constitutes the primary objective variable of the present study. (**b**) Plotted CA125-course of patient no. 164 throughout primary and recurrence therapy of a high-grade serous ovarian cancer high-grade serous epithelial ovarian cancer (HGSOC) patient undergoing platinum-based chemotherapy. The black lines demonstrate the respective growth rate estimates (g1 and g2) calculated applying the two-phased mathematical model, which were further evaluated for their predictive and prognostic values.

The tumor growth rate (g) and tumor decay rate (d) were calculated as previously described by Stein et al. using a biphasic mathematical function (f), by applying serial CA-125 measurements. The variable “t” describes the time in days after treatment commenced, “d describes the tumor decay, and “g” describes the respective tumor growth in the following equation: f(t)=exp(−d×t)+exp(g×t)−1 (Figure 1a) [7,8]. For every patient, growth “g1–g3” and decay “d1–g3” were automatically fitted using nonlinear least squares for first, second, and third recurrence, respectively. (Figure 1b) If after inspection the estimated decay–growth curve was deemed inappropriate, “g” and “d” were manually tweaked to better fit the data. For example, this was done for two patients to estimate “g” and “d” after the first-line treatment.

The resulting calculated logarithm of growth and decay rate estimates was further statistically evaluated as suggested by Stein et al. Based on the assumption that growth rate estimates quantify how fast respective tumors actually increase in size in-vivo, estimates were assessed for both their predictive and prognostic values at the timepoint of chemotherapy administration.

In the exploratory cohort, sequential CA-125 measurements were done from the primary surgery to the last follow-up, whereas this was done from the primary surgery to the first recurrence in the validation cohort. Therefore, the respective assessment of the 3rd and 4th lines of therapy was waived in the validation cohort.

Statistical analysis was performed using SPSS^®^ (IBM Corp. Released 2016. IBM SPSS Statistics for Windows, Version 24.0. Armonk, NY: IBM Corp.) for Windows and R 3.6.3 (R Core Team (2020). R: A language and environment for statistical computing. R Foundation for Statistical Computing, Vienna, Austria. URL https://www.R-project.org/ (accessed on 2 March 2021). Descriptive statistics of all patients in the exploratory and validation cohorts were computed. Categorical variables were described using percentages (%), and medians and interquartile ranges were used to describe continuous variables. The correlation between growth rate estimates and overall survival was described by Pearson’s correlation coefficient.

A logistic regression model was fitted to evaluate the growth rate estimate as a predictor of therapy response. A receiver operating characteristic (ROC) curve was computed and the optimal cut-off for the growth rate estimate to predict survival was defined by Youden’s J-statistics, as previously described [9]. This cut-off, as well as important confounders such as TFI > 6 months, age at time of therapy > 60 years, platinum-based therapy yes/no, and FIGO stage (2014) > FIGO IIIc were subsequently used in univariate analyses to determine their association with response to second-line, third-line, and fourth-line chemotherapy (using Chi-square tests), and overall survival at the time of secondary cytotoxic chemotherapy (log-rank tests). Subsequently, multivariable models for both endpoints (logistic regression models and Cox proportional hazard models) were fitted as deemed appropriate. For all effect estimates, 95% confidence intervals were computed. For all statistical tests, *p*-values < 0.05 were considered statistically significant. *p*-values serve only descriptive purposes; hence, no multiplicity corrections were applied.

## 3. Results

### 3.1. Description and Comparison between the Exploratory and Validation Cohorts

Table 1 depicts the characteristics of the patients in the exploratory and validation cohorts.

The key characteristics (patient’s age at the time of upfront debulking, residual tumor, FIGO stage, and follow-up) of the two cohorts were compared. To describe the effect of established prognostic factors in both cohorts, univariate and multivariate survival analyses were performed; FIGO stage (exploratory: *p* < 0.001, HR 3.12 [1.65–6.22]; validation: *p* = 0.01, HR 4.03 [1.76–9.22]); and no residual tumor after primary surgery (exploratory: *p* < 0.001, HR 2.90 [1.85–4.55]; validation: *p* = 0.01, HR 2.51 [1.24–5.07]) were independent predictors of OS. 

**Figure 2:**
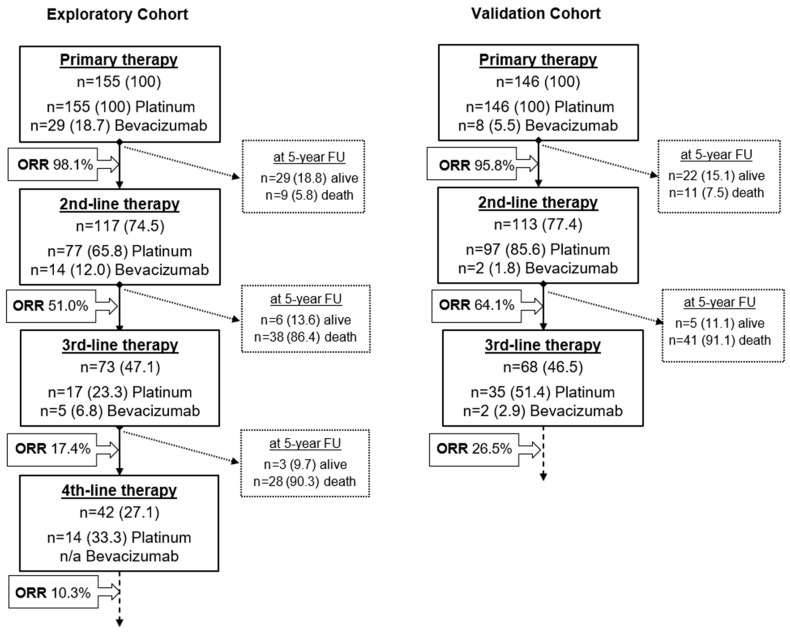
Flowchart depicting therapy courses and respective overall response rates (ORR) in comparison with platinum-based chemotherapy and bevacizumab application for both exploratory and validation cohort. Dashed boxes describe the survival status of patients who did not undergo a subsequent therapy line for any reason at a 5-year overall survival follow-up. The endpoint “death” equals patient death for any reason, including intercurrent disease. Numbers are given absolute and as percentages. n/a—data not available; ORR—overall response rate (complete and partial response).

A flowchart depicting therapy courses and respective response rates in comparison to platinum-based chemotherapy and bevacizumab application for both the exploratory and validation cohorts is given in Figure 2.

### 3.2. Growth Rate Estimate as a Predictor of Therapy Response 

**Figure 3:**
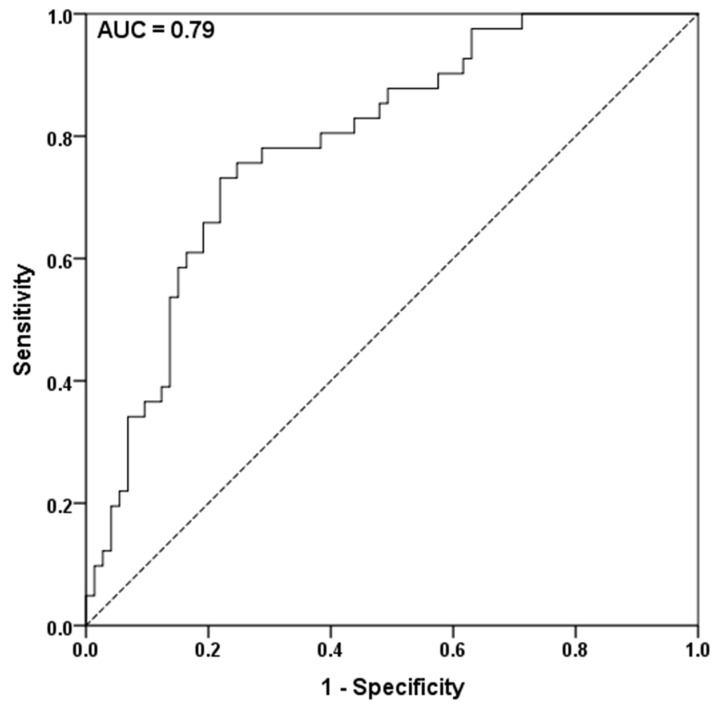
The area under the curve (AUC) receiver operating characteristic analysis to assess the predictive value of the logarithm of the growth rate constant in patients with recurrent HGSOC with the endpoint of complete or partial response to 2nd-line chemotherapy. Youden’s J-statistics was performed to define −3.32 as an optimal cut-off.

The area under the curve (AUC) of the receiver operating characteristic (ROC) curve was 0.790 (Figure 3). The ideal cut-off for the first recurrence after first-line chemotherapy treatment was −3.32, calculated with Youden’s J-statistics. The established cut-off was subsequently used for later recurrences.

Using a cut-off of −3.32, growth rate estimate (*p* = 0.003, OR 5.19 [1.73–15.58]), advanced patient age >60 years (*p* = 0.006, OR 5.32 [1.63–17.38]), and application of platinum-based chemotherapy (*p* < 0.001, OR 16.06 [4.48–53.29]) were predictive of the response to second-line cytotoxic chemotherapy in the multivariable analysis. TFI < 6 months was only predictive in the univariate analysis (*p* = 0.005, OR 1.73 [1.29–2.31]), but not in the multivariable analysis (*p* = 0.453, HR 1.95 [0.34–11.17]) (Table 2a). The same results were observed in the validation cohort (Table 2b).

The growth rate estimate (*p* = 0.017, HR 1.89 [1.06–3.37]), TFI > 6 months (*p* = 0.003, HR 1.71 [1.21–2.41]) and application of platinum-based therapy (*p* = 0.046, HR 1.94 [1.11–3.40]) remained predictive of a response to third-line chemotherapy only in the univariate analysis, whereas only application of platinum-based therapy (*p* = 0.010, HR 1.67 [0.87–3.19]) remained predictive of a response to fourth-line chemotherapy in the univariate analysis. There were no significant predictive factors for third- and fourth-line chemotherapy in the respective multivariable analyses.

The growth rate estimate retains its predictive value in homogenous patient subgroups, irrespective of the therapy-free interval

To confirm the predictive and prognostic value of the growth rate estimate in a homogenous cohort, a subgroup analysis was performed for all patients with a TFI > 6 months, who received platinum-based chemotherapy for the first recurrence and completed all therapy cycles (*n* = 69). In this subgroup, the growth rate estimate was associated with therapy response (*p* = 0.048, OR 2.46 [0.86–7.98]) and a prognostic factor for OS (*p* = 0.033, HR 3.31 [1.74–6.29]) in both univariate analyses. The results were not significant in the multivariable analyses. The growth rate estimate also retained its predictive ability in the subgroup of patients with a TFI < 6 months at first recurrence in the exploratory (*p* = 0.015, OR 5.00 [0.87–28.86], *n* = 11) and validation cohorts (*p* = 0.048, OR 4.0 [0.73–21.84], *n* = 9) in the univariate analysis. 

### 3.3. Growth Rate Estimate Is Independently Predictive of Overall Survival

**Figure 4:**
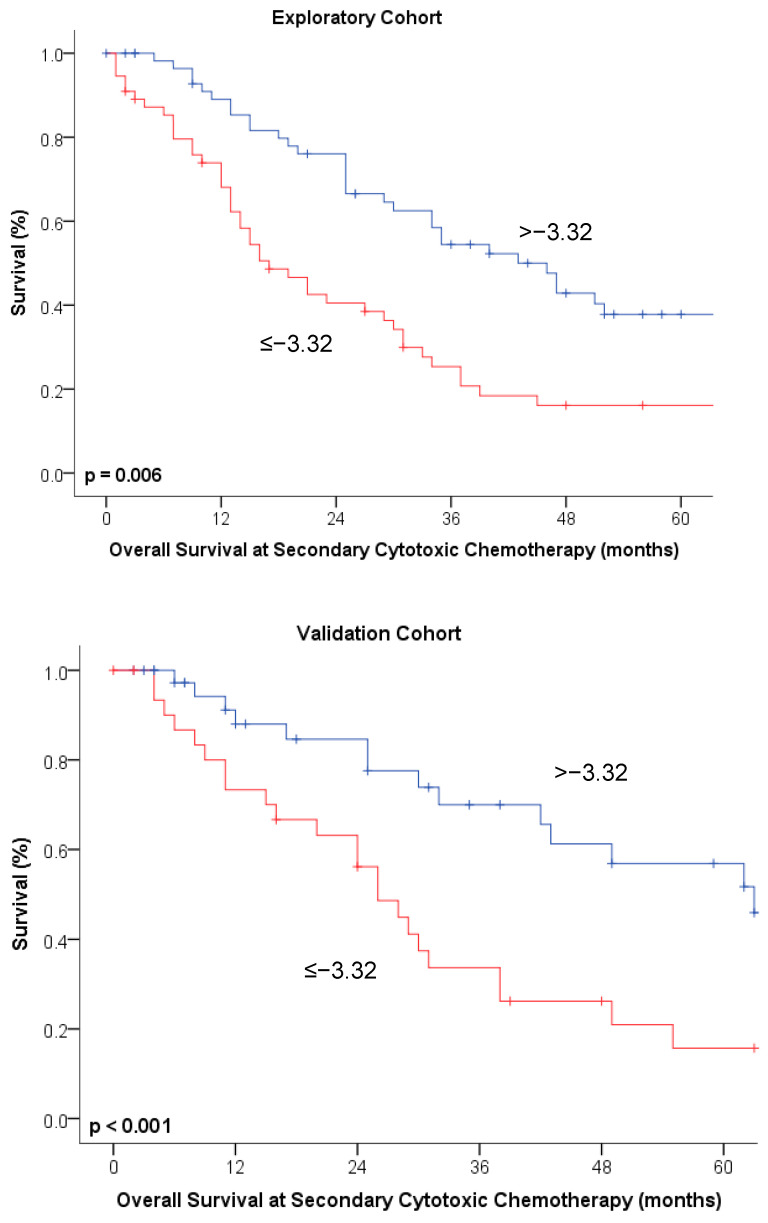
Kaplan–Meier curves depicting overall survival at the timepoint of secondary cytotoxic chemotherapy broken down by tumor growth rate estimates at an optimal cut-off of ≤/>−3.32 in both exploratory and validation cohorts.

Growth rate estimates for the 1st recurrence were significantly correlated with overall survival in both the exploratory (r = 0.39, *p* < 0.001) and validation cohorts (r = 0.44, *p* < 0.001). The growth rate estimate (*p* = 0.005, HR 1.92 [1.22–3.03]) and application of platinum-based therapy (*p* < 0.001, HR 2.34 [1.41–3.87]) were predictive of OS at the time of the first course of second-line chemotherapy in the multivariable analysis. TFI < 6 months was only prognostic in the univariate, but not in the multivariable analysis (*p* = 0.014) (Table 3a). The same results were observed in the validation cohort (Table 3b). Kaplan–Meier curves depicting the association between OS and growth rate estimates are given in Figure 4.

For the third-line chemotherapy (*n* = 73), growth rate estimate (*p* = 0.040), TFI > 6 months (*p* < 0.001), and platinum-based chemotherapy (*p* = 0.025) were prognostic factors only in the univariate analysis. For the fourth-line chemotherapy (*n* = 42), only use of platinum-based chemotherapy (*p* = 0.028) remained a prognostic factor in the univariate analysis. Respective multivariable analyses for responses to third- and fourth-line chemotherapy showed no significant results.

## 4. Discussion

Tumor growth rate estimates based on serial CA-125 levels are independent predictors of response to second-line chemotherapy and independent prognostic factors of patient survival after 2nd-line chemotherapy in patients with HGSOC. Tumor growth estimates outperformed the predictive and prognostic role of TFI < 6 months in the exploratory and validation cohorts.

The predictive value of tumor growth rates found in this study is in line with previously published results for renal cell carcinoma and prostate cancer. Stein et al. (2008) used the model in patients with prostate cancer in a clinical trial and subsequently validated it using fitting data from five prospective prostate cancer studies. They reported a higher efficacy of subsequent chemotherapy courses depending on incremental reductions in tumor growth estimates; they suggested that it was a cost-effective surrogate of patient survival for future clinical trials [7,8]. A prospective phase II trial of 81 patients with renal cancer confirmed the predictive value of the model for therapy response using the Response Evaluation Criteria in Solid Tumors (RECIST) criteria and also described its potential prognostic value [10]. The predictive and prognostic values of the model were recently validated in a prospective clinical study of 2353 patients from Project Data Sphere LLC (Cary, NC, USA). This study emphasized the growing clinical interest in the application of mathematical models in improving the understanding of the response to anticancer treatment [11].

This is the first adept study to evaluate the predictive and prognostic value of the model by Stein et al. in HGSOC. Tumor growth rate estimates may be promising surrogates to TFI in treatment decision making. Growth rate estimates may therefore allow the identification of patients in whom platinum reinduction earlier than 6 months after completion of the last cycle or beyond the first recurrence may be beneficial. 

This observation challenges earlier theories of clonal evolution and acquired common oncologic cellular resistance, which propose cytotoxic agents after a relapse. Although this assumption may be applied to other solid carcinomas, this study’s results may be explained by the confirmed exceptional chemosensitivity of HGSOC to platinum reinductions in two recent large clinical trials. First, the Mito-8 study, a phase III randomized trial tested whether intentionally extending the TFIp by avoiding platinum salts during the first relapse would extend survival. This hypothesis was clearly rejected, underlining the clinical benefits of early platinum reinduction [12]. Second, INOVATYON, a phase III randomized trial, failed to demonstrate an improvement in OS after a trabectedin/pegylated liposomal doxorubicin (PLD) combination instead of carboplatin/PLD for platinum-sensitive HGSOC recurrence, confirming the efficacy of platinum-based regimens in recurrence [13].

In line with this, preclinical longitudinal studies investigating the molecular adaptions of HGSOC cells after platinum therapy failed to consistently demonstrate specifically selected mutations attributable to particular cellular platinum resistance mechanisms, which may help to identify patients unfit for platinum reinduction. Genomic profiling before and after treatment showed near-identical genomic landscapes without distinct mutational adaptions [14,15,16]. Most mutations appear to exist before primary therapy and persist throughout the treatment with platinum, suggesting that several clones survive therapy without accumulating distinct mutations that are attributable to particular chemoresistances [17]. 

Recent evidence of the possible benefits of platinum reinduction outlines the urgent necessity of defining strong predictors of therapy response to allow more personalized treatment decisions, and subsequently improve survival.

Numerous mathematical models have been proposed to date as potential aids in clinical decision making such as nomograms to interpret established biomarkers more comparatively or more complex risk prediction models such as the broadly validated IOTA ADNEX [18,19]. Of note, mathematical models also face methodological limitations which have to be considered before application: Apart from rigorous broad clinical multicenter validation, sensitivity and model error analyses should always be performed before application at a new center to account for possible systematic confounders not depictable by the model design. Moreover, mathematical models may only address specific questions and may be limited in scale as inclusion of more factors may require both methodologically and computationally intensive numerical integration. In case of the present model fitted for HGSOC patients, data of two patients had to be manually tweaked as the model failed to automatically account for prolonged remission times. This issue was already addressed by the original publication of Stein et al. [20,21].

This study had some limitations. First, as is typical of retrospective studies, its lack of random patient assignment and selection and possible erroneous data acquisition may diminish its clinical value. However, its large sample size and long follow-up period may have ensured the generalizability of our data due to a change in treatment regimens and existing evidence. Therefore, data from a larger prospective multicenter cohort were adopted as a validation cohort to minimize the associated bias. Since observations could be independently confirmed in both cohorts, it may be assumed that our study findings are generalizable. It is to be noted that growth rate estimates retained both predictive and prognostic value for 3rd-line chemotherapies only in univariate analyses and failed to prove significant for 4th-line chemotherapies. Whereas these results may be considered to be expected due to dwindling patient numbers in respective cohorts, resilient results would be interesting for this particular subset of patients, which the present study failed to provide. Moreover, bevacizumab application may be considered a confounding factor for survival analysis as results of GOG218 and ICON7 trials indicated survival benefits for patients, including those staged FIGO 4. As the utmost part of patients, however, received primary treatment before the results of the GOG2018 and ICON7 were published, only a subset of included patients was treated with bevacizumab in the primary treatment setting [22,23]. Respective treatment differences between exploratory and validation cohort may be explained by slightly different timepoints of chemotherapy administration (median timepoint of first chemotherapy administration March 2008 for the exploratory cohort and April 2006 for the validation cohort) as well as by different national treatment standards. Nevertheless, our study findings should be interpreted as exploratory, requiring prospective validation in larger cohorts before their application in clinical routine. 

## 5. Conclusions

Tumor growth rate estimates based on serial CA-125 levels are independent predictors of therapy response and prognosis of recurrence in HGSOC, irrespective of TFI. Thereby, tumor growth rate estimates may provide a novel, easily quantifiable, and cost-effective biomarker of chemotherapy response and tumor recurrence, which may complement the TFI in therapy decision making and improve personalized treatment strategies. Further clinical validation in larger patient cohorts or within the frame of prospective trials is warranted.

## Figures and Tables

**Table 1:** Descriptive characteristics. Patients treated at the Medical University of Vienna comprised the exploratory cohort, while patients treated at partner centers of the Ovarian Cancer Therapy–Innovative Models Prolong Survival (OCTIPS) consortium comprised the validation cohort.

Parameter	Exploratory Cohort	Validation Cohort
Number of patients	155	146
Age at surgery (years)	58.0 (49.0–66.0)	57.0 (48.8–64.3)
FIGO stage (2014)		
II	6 (3.9%)	5 (3.4%)
III	132 (85.2%)	123 (84.3%)
IV	17 (11.0%)	18 (12.3%)
Residual tumor after primary surgery		
Yes	48 (30.8%)	50 (34.2%)
No	107 (68.6%)	96 (65.8%)
CA-125 at primary surgery (kU/L)	558.0 (221.5–1491.3)	504 (140.5–1301.0)
Time to nadir (days)	103.5 (73.3–161.3)	not available
Log growth rate estimate at 1st recurrence	−3.5 (−4.3–−3.1)	−3.4 (−4.1–−3.0)
Log decay rate estimate at 1st recurrence	−1.3 (−1.5–−1.1)	−1.0 (−1.1–−0.9)
Status at last follow-up		
Alive	38 (24.5%)	42 (28.8%)
Progression	4 (2.6%)	not available
Intercurrent death	26 (16.8%)	18 (12.3%)
Cancer-related death	87 (56.1%)	78 (53.4%)
Duration of follow-up (months)	53.0 (27.0–85.0)	46.0 (28.8–71.5)
TFST (months)	15.0 (8.0–25.5)	14.5 (7.0–23.0)
Available	117 (75.5%)	113 (77.4)
Not available	38 (24.5%)	33 (22.6)
TSST (months)	7.0 (2.5–11.5)	8.0 (3.0–13.0)
Available	73 (47.1%)	68 (46.6%)
Not available	82 (52.9%)	78 (53.4%)
TFI 2nd to 3rd recurrence (months)	5.0 (1.0–10.0)	4.0 (1.0–9.0)
Available	42 (27.1%)	45 (30.8%)
Not available	113 (72.9%)	101 (69.2%)

Values are presented as median (interquartile range) or number (%). CA-125, cancer antigen 125; FIGO, International Federation of Gynecology and Obstetrics; TFST, time to first subsequent therapy; TSST, time to second subsequent therapy; TFI, therapy-free interval.

**Table 2:** (**a**–**b**) Predictive factors for response to second-line cytotoxic chemotherapy for HGSOC recurrence in the exploratory (2a) and validation cohort (2b). The endpoint of therapy response is classified as either complete or partial response after completed chemotherapy.

**2a. Parameters** **Exploratory Cohort**	**Response to Second-Line Chemotherapy**			
	**Univariate Analysis**		**Multivariable Analysis**	
	***p*-Value**	**OR (95% CI)**	***p*-Value**	**OR (95% CI)**
Log growth rate constant >−3.32	<0.001	2.57 (1.60–4.12)	0.003	5.19 (1.73–15.58)
Therapy-free interval >6 months	0.005	1.73 (1.29–2.31)	0.453	1.95 (0.34–11.17)
Age at time of therapy >60 years	0.03	1.35 (1.04–1.74)	0.006	5.32 (1.63–17.38)
Platinum-based therapy yes/no	<0.001	5.39 (2.92–9.95)	<0.001	16.06 (4.48–53.29)
FIGO stage (2014) > FIGO IIIc	0.055	1.69 (0.86–3.34)	-	-
**2b. Parameters** **Validation Cohort**	**Response to Second-Line Chemotherapy**			
	**Univariate Analysis**		**Multivariable Analysis**	
	***p*-Value**	**OR (95% CI)**	***p*-Value**	**OR (95% CI)**
Log growth rate constant >−3.32	0.008	1.60 (1.10–2.33)	0.03	3.67 (1.13–11.86)
Therapy-free interval >6 months	0.025	1.81 (0.95–3.47)	0.661	1.48 (0.26–8.46)
Age at time of therapy >60 years	0.03	7.58 (1.11–51.66)	0.056	10.15 (0.94–109.25)
Platinum-based therapy yes/no	<0.001	2.65 (1.51–4.65)	0.016	5.79 (1.40–24.05)
FIGO stage (2014) > FIGO IIIc	0.048	1.68 (0.89–3.18)	-	-

CI: Confidence interval, FIGO: International Federation of Gynecology and Obstetrics, OR: Odds ratio.

**Table 3:** (**a**–**b**) Univariate and multivariate analysis of prognostic factors of overall survival at the time of secondary cytotoxic chemotherapy for high-grade serous ovarian cancer recurrence in both the exploratory (3a) and validation (3b) cohorts. Endpoint was patient death irrespective of documented cause.

**3a. Parameters** **Exploratory Cohort**	**Prognostic Value for Second-Line Chemotherapy**			
	**Univariate Analysis**		**Multivariable Analysis**	
	***p*-Value**	**HR (95% CI)**	***p*-Value**	
Log growth rate constant > −3.32	0.006	2.13 (1.37–3.32)	0.005	1.92 (1.22–3.03)
Therapy-free interval > 6 months	0.014	1.93 (0.96–3.87)	0.524	1.29 (0.59–2.82)
Age at time of therapy > 60 years	0.005	1.82 (1.17–2.85)	-	-
Platinum-based therapy yes/no	<0.001	2.66 (1.69–4.17)	0.001	2.34 (1.41–3.87)
Bevacizumab received yes/no	0.934	1.02 (0.60–1.76)	0.504	1.25 (0.65–2.38)
No primary surgery residual tumor (R0)	0.045	1.46 (0.93–2.29)	-	-
FIGO stage (2014) > FIGO IIIc	0.595	1.32 (0.66–2.65)	-	-
**3b. Parameters** **Validation Cohort**	**Prognostic Value for Second-Line Chemotherapy**			
	**Univariate Analysis**		**Multivariable Analysis**	
	***p*-Value**	**HR (95% CI)**	***p*-Value**	
Log growth rate constant >−3.32	<0.001	3.31 (1.74–6.29)	0.002	2.90 (1.50–5.59)
Therapy-free interval >6 months	<0.001	1.44 (0.73–2.81)	0.642	1.13 (0.83–1.79)
Age at time of therapy >60 years	<0.001	1.58 (1.01–2.48)	-	-
Platinum-based therapy yes/no	0.001	2.10 (1.30–3.39)	0.015	2.23 (1.17–4.23)

CI: Confidence interval, FIGO: International Federation of Gynecology and Obstetrics, HR: Hazard ratio.

## Data Availability

Data of the exploratory cohort are available on request from the corresponding author. As data of the validation cohort were obtained from our partner centers, they are only available after respective permission.
